# Promoting participation in healthcare situations for children with JIA: a grounded theory study

**DOI:** 10.3402/qhw.v11.30518

**Published:** 2016-05-10

**Authors:** Britt-Mari Gilljam, Susann Arvidsson, Jens M. Nygren, Petra Svedberg

**Affiliations:** 1 Region Halland, Halmstad Hospital, Sweden; 2 School of Social and Health Sciences, Halmstad University, Halmstad, Sweden

**Keywords:** Children, healthcare, participation, constructivist grounded theory, juvenile idiopathic arthritis

## Abstract

Children's right to participate in their own healthcare has increasingly become highlighted in national and international research as well as in government regulations. Nevertheless, children's participation in healthcare is unsatisfactorily applied in praxis. There is a growing body of research regarding children's participation, but research from the children's own perspective is scarce. The aim of this study was thus to explore the experiences and preferences for participation in healthcare situations among children with juvenile idiopathic arthritis (JIA) as a foundation for creating strategies to promote their participation in pediatric healthcare. Twenty children, aged 8 to 17 years, with JIA were interviewed individually and in focus groups. In order to increase the children's opportunities to express their own experiences, different interview techniques were used, such as draw-and-tell and role play with dolls. The analysis was conducted with a constructivist grounded theory. The result explores children's perspective of influencing processes promoting their participation in healthcare situations. The core category that emerged was, “Releasing fear and uncertainty opens up for confidence and participation,” and the categories related to the core category are, “surrounded by a sense of security and comfort,” and “strengthened and supported to become involved.” In conclusion, the knowledge gained in this study offers new insights from the perspective of children themselves, and can constitute a valuable contribution to the understanding of necessary conditions for the development of specific interventions that promote participation among children in healthcare situations.

Participation, defined as taking active part in consultations with healthcare professionals (Fumagalli, Radaelli, Lettieri, & Masella, 2015), has increasingly become a part of common practice in healthcare as a result of qualitative research and government regulation (Shay & Lafata, 2014). In spite of this development, and the existence of laws and regulations concerning children's rights (Department of Health and Children, 2000; Swedish-Parlament, 2014; United Nations Convention on the Rights of the Child, 1989), those that encompass involvement and participation in healthcare are unsatisfactorily applied in praxis (Clark et al., 2009; Virkki, Tolonen, Koskimaa, & Paavilainen, 2014). Research shows that the abilities of children to express their needs and wishes in healthcare is restricted, due to their dependence of others (Mårtenson & Fägerskiöld, 2008) and a tradition of communication that is focused on healthcare professionals and parents (Butz, Walker, Pulsifer, & Winkelstein, 2007; Young, Moffett, Jackson, & McNulty, 2006). Consequently, children do not often experience being able to participate and feel neglected in healthcare situations (Coyne & Kirwan, 2012; Peña & Rojas, 2013), even though they desire to be involved in their own healthcare (Coyne, 2006; Lipstein, Muething, Dodds, & Britto, 2013). The reasons may be that parents often respond on their children's behalf (Butz et al., 2007), and healthcare professionals are unaccustomed to valuing children's own views and lack the tools to implement children's participation (Coyne, 2008; Oruche, Downs, Holloway, Draucker, & Aalsma, 2014; Ruhe, Wangmo, Badarau, Elger, & Niggli, 2014). The consequences of insufficient participation are particularly severe for children with chronic diseases as their needs for extensive care place greater demands on meaningful interaction with healthcare professionals (Sällfors & Hallberg, 2009).

Children who suffer from juvenile idiopathic arthritis (JIA) have to deal with functional impairment, pain, medication, and several examinations during healthcare visits (Eyckmans, Hilderson, Westhovens, Wouters, & Moons, 2011; Sällfors & Hallberg, 2009). JIA is a chronic disease with an uncertain prognosis and intense treatment with uncertain outcomes and a high risk of severe side effects (Bertilsson, Andersson-Gäre, Fasth, & Forsblad-d'Elia, 2012; Beukelman et al., 2011; Eyckmans et al., 2011; Sällfors & Hallberg, 2009). This means that these children have to understand and manage many issues related to their own disease (Eyckmans et al., 2011), and they have a large number of contacts with healthcare professionals over a long period of time. Nevertheless, research regarding participation in healthcare among children with JIA is scarce. One study describes that female adolescents with JIA emphasize the importance of participation in all phases of pain management and healthcare (Sällfors & Hallberg, 2009). As they get older, many children with chronic diseases want to have a more prominent role in discussions with healthcare professionals, for which they need skills and knowledge (Lipstein et al., 2013; Wyatt et al., 2015). Children who do not understand what is happening to them or do not feel that they have an active part in healthcare situations are prone to feelings of fear, anxiety, and stress, and are more likely to continue to be inadequately prepared for ongoing examinations and procedures (Coyne, 2006; Runeson, Hallström, Elander, & Hermerén, 2002). It is thus essential to gain a greater understanding of children's own perspective on how their participation can be promoted in healthcare. This knowledge can lead to a more qualitative and appropriate care for children with JIA. The aim of this study was thus to explore experiences and preferences for participation in healthcare situations among children with JIA as a foundation for creating strategies to promote their participation in pediatric healthcare.

## Materials and methods

The present study was conducted with an explorative qualitative design based on constructivist grounded theory (Charmaz, 2014). A researcher who uses this theory attempts to understand experience and its meaning in the same way as the participants (Charmaz & McMullen, 2011).

### Participants

The children were recruited from two pediatric clinics in southern Sweden. The inclusion criteria were children aged between 8 and 17, having a diagnosis of JIA for at least 2 years, and having undergone at least two intra-articular corticosteroid injections (IACI). Children with delayed development and children who did not understand Swedish were excluded. A letter was sent to the parents from the child's rheumatic nurse. The first author then contacted the parents by telephone to inform about the study's purpose and outline, and invite the child to participate. The parents and children who were interested in participating in the study received written information about the study. In order to achieve diversity in the sample, the children who were invited to participate were varied in age, gender, and contact with different hospitals (Table I), differences that were found at both hospitals. Out of 36 children, 8 girls and 8 boys declined to participate in the study. Of these 16 children, three parents decided not to participate in the study without consulting the child, in the belief the child was already in a stressful position and needed to be protected.

**Table I:** Sociodemographic characteristics of the children (*n*=20).

	*n*	%
Gender		
Male	5	25
Female	15	75
Age mean (range)	12.5 (8–17)	
Living		
With both parents	18	90
With one parent	2	10
Time from diagnosis		
2 years	1	5
3–5 years	8	40
>5 years	11	55
Intra-articular corticosteroid injections (IACI)		
2 times	5	25
3–5 times	10	50
>5 times	5	25
Experienced pain relief under IACI		
No pain relief	2	10
Per oral analgesic/sedative	2	10
Nitrous oxide	5	25
Anesthesia	6	30
Nitrous oxide+anesthesia	3	15
No pain relief+anesthesia	2	10
Ongoing JIA treatment with injections		
Yes	8	40
No	12	60

### Data collection

A total of 20 children were interviewed during a period of 8 months in 2013 and 2014; 9 children were in three focus groups and 11 children were in individual interviews (Table I). Three children from the focus groups who were interviewed individually a second time were asked ask further questions in order to obtain more profound data. The focus groups were gender-mixed and divided into age groups, with maximum 2-year differences between the children in each group. Each focus group consisted of three children and two researchers, and lasted 2 h with a small pause. The focus group interviews were carried out by the first author, and the second author observed and wrote notes. The first author carried out all the individual interviews. All the focus group interviews began with a short refreshment break for the younger children and the parents, who also attended in order to help the children feel comfortable in the interview situation. The parents then remained close by while the interviews took place, and a child could withdraw from the interview if desired so. The interviews started with the open question, “Can you tell me about a situation when you got an IACI?” All the children had been exposed to this procedure at least twice. The younger children were first asked to draw a situation from an IACI or from the waiting room before they received an IACI and then to talk about the drawing (Larsson & Lamb, 2009). The younger children in the focus groups also used dolls in a role play about a situation describing a doll undergoing an IACI. Open-ended questions were asked during the interviews to elicit narrative answers from the children about the issue. The interviewer then followed up with probing questions when relevant, for example, concerning how the children preferred to be treated or why they preferred particular procedures. In constructivist grounded theory, the researcher interprets how the participants construct their realities and meanings (Charmaz, 2014). All the interviews were videotaped to record facial expressions and body language. In accordance with constructivist grounded theory, the data collection and data analysis were performed simultaneously and continued until no new data emerged (Charmaz, 2014). The analysis of one interview led to more profound issues in the next interview.

### Data analysis

The first author transcribed the 17 interviews verbatim immediately after each interview, and a preliminary analysis was initiated. The authors studied the transcribed interviews several times in order to be able to understand what the children really meant. The first author wrote memos during the data collection and data analysis. Events captured on the film and these memos were also included in the analysis. BG and SA performed the analysis with continuous discussions with the other two authors. During the initial coding, the interviews were analyzed line by line, coding phrases that defined what the data concerned. The authors attempted to see actions and social processes in each piece of data and conceptualize them. In the next phase, focused coding, categories were elucidated in the form of actions and processes. The data were constantly compared with previously coded incidents. A theoretical sampling was made when some children from the focus groups were individually interviewed a second time. The purpose was to elaborate the categories until no new properties emerged. Constant comparisons were performed between the data, the codes, and the categories, back and forth. A core category emerged with an interpretive understanding from the concepts forming the categories (Charmaz, 2014). The core category with related categories are described and illustrated by quotations from the interviews in the result section.

### Ethical considerations

The study was approved by the Regional Ethical Review Board in Lund (Dnr: 2012/821). The children's participation was discussed with both parents and the children. They were informed that they could withdraw from the study at any time without justifying the reason. Informed consent from both children and parents were a prerequisite for participation. All data were treated confidentially. The interviews were coded to protect the anonymity of the children.

## Results

**Figure 1:**
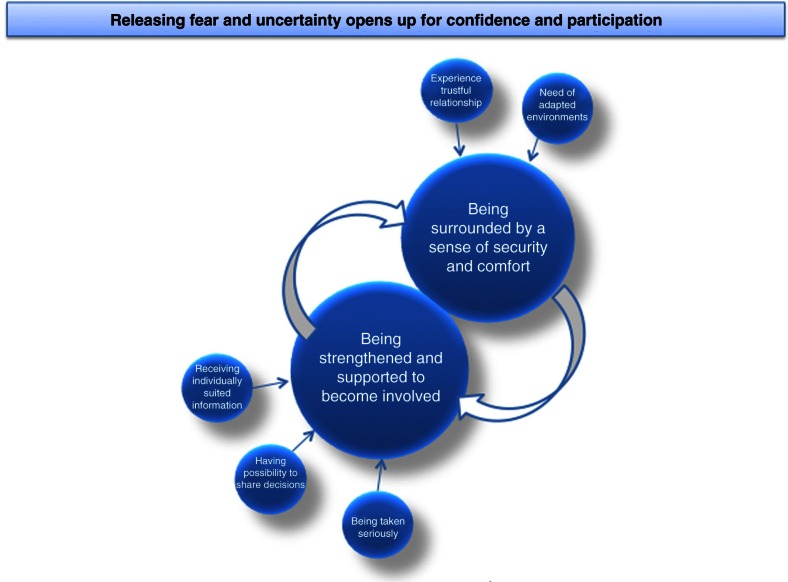
Illustration of the core category, categories, and related subcategories.

The core category that emerged in the analysis was, *“*releasing fear and uncertainty opens up for confidence and participation.” The children's feelings of fear and uncertainty influenced and inhibited their possibilities for participation in healthcare situations. In order to promote the children's participation, they needed to be *surrounded by a sense of security and comfort* and be *strengthened and supported to be involved* (see Fig. 1).

### Releasing fear and uncertainty opens up for confidence and participation

Avoiding fear and being calm were the most important issues for the children in all the healthcare situations mentioned in all interviews, regardless of age. It also emerged that many of the children felt uncertain in healthcare situations, not knowing how to act or what was expected of them. The children's fear and uncertainty created a lack of trust and feelings of stress that inhibited participation. In order to promote the children's participation, they needed to *be surrounded by a sense of security and comfort* for being able to feel calm in healthcare situations. This process was influenced by trustful relationships with healthcare professionals and environments modified for pediatric healthcare. In order to dare to participate in healthcare situations, the children also needed to *be strengthened and supported to become involved*. They felt that they were strengthened when they were being taken seriously, were given information individually, and allowed to share decision making. They felt they were treated as unique individuals when they were respected and their voices heard. In order to give the children a feeling of control over the situation, and the self-confidence and courage to ask questions, healthcare professionals supported them by continuously asking the children for their opinions and making sure they understood both the information and what was going to happen. These actions generated a sense of security and strengthened the children's ability to participate. Their experiences of trust and their ability to concentrate on what was said was strengthened when their fear and uncertainty was released, resulting in the children daring to ask questions and communicate with healthcare professionals. In order for the children to participate in healthcare situations, which formed the categories, it was thus important for them to feel that they both were *surrounded by a sense of security and comfort* and *strengthened and supported to become involved*.

### Being surrounded by a sense of security and comfort

Children's experience of being surrounded by a sense of security and comfort was an important dimension of promoting their participation in healthcare situations. Experiences of security and comfort in the healthcare situation depended on the degree of trust the children had in their relationships with the healthcare professionals and how the environments were adapted to pediatric healthcare.

The environment, such as examination, treatment, and waiting rooms, created distraction as well as comforting feelings when they were adapted to the children's ages and situations. The environment, in terms of sound and interior design, as well as the amount of waiting time, influenced the children's experiences. Colorless and sterile rooms were experienced as boring and threatening. The children wanted colorful rooms with windows and pictures on the walls. A 17-year-old girl stated, “It shouldn't be a very sterile room, just bare, white walls. Like when I was lying there and got it (IACI) and I had the universe above me (a picture of space on the ceiling), it felt good because then I had the universe to focus on. So you can get away a little” (Child 1, individual interview, first time). Access to toys and other forms of distraction, such as an aquarium in the waiting room, were important. Having music during treatments calmed some children. These and other forms of distractions were useful ways for the children to shed their fears for a while, feel more comfortable in the situation, and participate in the healthcare situation. The younger children spoke of the importance of reduced waiting time in healthcare environments, which promoted feelings of security and prevented worry. A 10-year-old girl's experience of the waiting time was, “You don't want to just sit in the waiting room and only wait and wait and wait and wait, you want them to be ready quicker. I was very nervous you see” (Child 1, focus group 2).

The children wanted healthcare professionals with gentle voices and pleasant background sound during examinations and treatments. They felt more relaxed when healthcare professionals talked and moved slowly, whereas disturbing noises and stressed-out healthcare professionals triggered fear in the children. It was important that only a few people remained in the room before and during an IACI to create a sense of security, irrespective of whether the child was receiving analgesics or anesthesia. A girl, 12 years old, receiving anesthesia described her experience, “there was so much happening around you, people running around and checking … it feels as if you're dying” (Child 8, individual interview, first time). The IACI procedure was very frightening in itself and having many people in the room increased the level of stress and made the children feel that they were undergoing a serious operation. When healthcare professionals were talking with the children during the IACI, preferably with a sense of humor, they felt calm and secure, and were helped through the procedure. The children received effective pain relief when they inhaled nitrous oxide during the IACI, while a nurse told a story. A voice telling a story was enthralling and relaxing, and one girl, 10 years old, said, “the nurse told a good story, it was about horses, and we were tumbling in the hay, and then … I was nearly falling asleep, I became just so relaxed, and I laughed a lot” (Child 2, individual interview, second time).

The children described feeling assured by always meeting the same healthcare professionals. These meetings promoted trust in these relationships and the children felt more understood as persons and for their situation. They felt relieved about not having to repeat their story over and over again. When encountering a new, unfamiliar healthcare professional, the children withdrew, watched, and waited. They did not dare to be active and participate, and did not ask questions. A girl, 10 years old, said, “So, of course I want the old one (the doctor), that it's the one I have now, because, like … new ones, they don't, sort of, know me, how I am inside and so on. I don't know if he is kind or if he is, sort of, harsh, or so” (Child 2, individual interview, second time). On the contrary, some children appreciated a variety of healthcare professionals and thought it was great to have a new doctor validate the treatment. Most of the older children admired their doctors for their knowledge and power. Most girls preferred meeting women as healthcare professionals as they did not like disrobing in front of men.

The children wanted to be met and treated with respect as individuals, and not just treated in relation to their age. They pointed out that all people are different, and how important it is to be treated as a unique person by healthcare professionals. They wanted friendly conversations with healthcare professionals about personal topics, such as sports, competitions, pets, travels. A 15-year-old girl said, “we talk about how I feel and what I'm doing in my free time and such … that's important, you can't be obsessed with your disease” (Child 3, individual interview, second time). If a healthcare professional remembered something from a previous conversation, the child felt that she or he was important. The children appreciated healthcare professionals who built personal friendships with them, which promoted feelings of security and opened up for the ability to participate.

Many children felt uptight and anxious before visiting the hospital. They were afraid the visit might end up in bad news of how the disease was progressing, or that they would need to undergo needle procedures. These emotions blocked the children and inhibited opportunities for participation. The children noted it is important for the healthcare professionals to tell the truth. Many children had experienced healthcare professionals telling them that an IACI would not hurt. The children then felt deceived and betrayed because the treatment was very painful, which led to distrust and skepticism about healthcare professionals in general. One 17-year-old girl said, “Just a little pain, or no pain at all, means it hurts a lot. I have learned, so I don't trust them” (Child 1, individual interview, first time). A 10-year-old girl said, “… after that it was much harder. The first time, I really didn't think it would hurt, because the doctor said so and I trusted him” (Child 2, individual interview, second time).

### Being strengthened and supported to become involved

The children's experience of being strengthened and supported to become involved in healthcare situations was reported as an important dimension of promoting their participation. Feelings of uncertainty emerged when the children did not know what would happen or how they were supposed to act in care situations. In order to counteract this, healthcare professionals could strengthen and support the children in a number of ways: taking them seriously, giving them individually based information, and promoting possibilities for them to share in decision making by carefully listening to them, repeatedly informing them, and involving them in consultations and decisions. All the children wanted the healthcare professionals to ask them how active they wanted to be in healthcare situations. Some, both the younger and older, wanted to be more actively involved than they already were.

When healthcare professionals took the children seriously, asked for the children's opinions, and carefully listened to them, feelings of influence and power emerged, and opportunities to participate were promoted. The children thought it was important to ask every individual child about their opinion and how they wanted things to be in healthcare situations, because each child is unique. A 17-year-old girl said, “Maybe they (healthcare professionals) can ask us how we want it to be, because I think, you don't get as many questions as maybe you need to, not so many say how they want it to be, because they perhaps don’t dare. The doctors think they do the right things, because they are doctors. But it’s not always like that. It’s different from person to person ….” (Child 6, individual interview, first time). It was important for the children to have their voices heard and to be believed, and for the healthcare professionals to show an interest in them. Some children were mostly quiet and had not thought about sharing their wishes, for example, an 8-year-old boy, “I would prefer to take my blood tests in the finger, that doesn't hurt at all, and you don't have to wait so long, for the EMLA. (Researcher asked: Have you asked if you can have it taken in your finger?) No” (Child 4, individual interview, first time).

When the healthcare professionals believed what the children said, the children felt supported and secure, and their self-esteem increased. Most healthcare professionals were described as good listeners who took the children's opinions into account, which promoted participation. Children noticed whether the healthcare professionals paid full attention or not, and whether they listened empathically. A 15-year-old girl described it, “… you can see by their body language if they really are listening to you. They should seem to be interested in what I have to say. Or else (shaking her head), else I don't trust them” (Child 3, individual interview, first time).

When children were invited to participate in conversations with healthcare professionals, their self-confidence grew and they felt strengthened, supported, and respected. Some children left decision making to their parents and doctors. This behavior was not associated with the children's age or gender. Even if they, just then, did not want to share any decision, they wanted to feel the possibility existed that they could do so. The decision that children most frequently left to the doctor was medication strategies. The decision making that children wanted most often concerned the administration of medicines, how to perform an IACI or a blood test, and who should be giving injections at home. This 17-year-old girl said, “I could choose to take the pills and see if it worked, or choose the new medicine, so I choose to test the pills, because it‘s unnecessary to take injections every week if you don't have to” (Child 1, individual interview, first time). Receiving information about the medication in comprehensible words and conveying the medical effect was important. A 15-year-old boy said, “Chemotherapy entails after all quite powerful medication … those who have cancer take them … they'll break you down in the end” (Child 10, individual interview, first time). Some doctors allowed children to decide when a needle should enter during an IACI; this made children feel that they were encouraged to participate a lot. Feelings of having their participation stimulated also occurred when they were given choices, such as suggestions about when and how an IACI should be performed.

The children requested to be repeatedly informed on an individual basis, which could be performed by healthcare professionals drawing or showing on dolls, in order to assure that the children had really understood correctly. Both younger and older children sometimes experienced difficulty receiving and understanding information during meetings with healthcare professionals. A 13-year-old girl said, “Sometimes when we sit and talk, I usually don't understand, so when we are on the way out I ask mom, like, what they had talked about” (Child 1, individual interview, second time). There were also children who did not care when they did not understand, as if resigned about understanding and participating. For example, one 15-year-old boy said, “(Researcher asked: Do you ask if there is anything you don't understand?) No, actually not. (Researcher asked: Do you let it be, or do you ask your parents then?) No, I just… carry on talking. (Researcher asked: You just leave it?) Yes” (Child 10, individual interview, first time). When they left the hospital, some children talked to their parents and some searched on the Internet for answers to their questions, in order to get explanations in a calm setting, without stress. To promote children's understanding and feelings of being supported, the children wanted to be well informed and able to understand the progress of the disease, what the illness could lead to, and the treatment and its side effects. When the children felt well informed, it was easier for them to accept bad news, such as the need for an IACI. A 10-year-old girl described how she used to coach herself before an IACI, “I think for myself: you can do it, if you don't do it, you would have it …., you would never ever be able to ride a horse again in your whole life. I think of things like this. … so I had to, because I don't want stop riding!” (Child 2, individual interview, second time). If the children needed anesthesia, it was important for them to be informed about how they would feel and where they would be when they woke up. Those who were monitored after an IACI asked for explanations about what the machine indicated and what was happening when scary-sounding alarms went off. This helped the children to be more prepared, strengthened, and secure. When the children understood the whole picture, they became more daring about asking questions about minor issues that they did not understand.

## Discussion

The study explores children with JIA's perspective of influencing processes to promote their participation in healthcare situations. The results highlight the importance of releasing children's fear and uncertainty in order to promote feelings of confidence and increase their possibility of participation in healthcare. The children's ability to take an active role in their own care increases if they experience security and comfort in encounters with healthcare professionals. This in turn affects children's feelings of influence and power, increases their self-confidence, and better prepares them to face healthcare situations. This result can be used as a foundation for creating strategies to promote participation in pediatric healthcare.

The result in this study applies the “pathways to participation model” (Shier, 2001), which is a practical model for healthcare professionals to understand how to promote different levels of children's participation in healthcare (Shier, 2001). “The pathways to participation model” is intended to be used with children aged 8–12 years and consists of five levels: (1) children are listened to, (2) children are supported in expressing their views, (3) children's views are taken into account, (4) children are involved in decision making, and (5) children share power and responsibility for decision making. In the present study, the children expressed, in accordance with levels one to three of the model, that they want to be listened to, receive information based on their own personal needs, have the possibility of expressing their views, and know that their opinions will be taken into account in every healthcare situation. The children also wanted to have the opportunity for active participation in decision making, which is in line with level four. In the fifth level in the model, the children have actual power over decisions; however, the children in the present study did not explicitly describe this as being important. Their main objective was more focused on the healthcare professionals providing them with the opportunity to involve in decision making if they wanted to do so. They wanted the healthcare professionals to ask them how active they wish to be in any particular healthcare situation. Interventions that attempt to promote children's shared decision making in healthcare have increased over the last 5 years; however, the positive effects have only been found in the increased knowledge of parents and a reduction in decisional conflicts, but not concerning the children themselves (Wyatt et al., 2015). Many healthcare professionals want to promote participation of children in healthcare decisions (Ruland, Starren, & Vatne, 2008; Vaknin & Zisk-Rony, 2011), but healthcare organizations and limited financial resources make it difficult (Virkki et al., 2014), which means that ambitions are not always realized. This may indicate the need to implement specific methods that can help healthcare professionals in supporting children's participation in their own care. A method used in pediatric care, which has generated improvements in the communication between children and healthcare professionals, is Sisom (Ruland et al., 2008), an interactive digital tool in which the child travels through a world of islands and expresses how he or she feels (Vatne et al., 2012). The child's estimation in Sisom laid the foundation for communication between the child and the healthcare professional, with the objective of ensuring that the staff is working from a child's perspective.

In order to open up for participation in healthcare situations, the children in the present study explicitly expressed the need to be surrounded by a sense of security and comfort. Experiences of security and comfort in the healthcare situation depended on a trusting relationship between the child and the healthcare professional, and on an environment that had been adapted to the children's preferences. A trustful relationship was created through the healthcare professional's ability to be friendly, have a positive attitude, and show interest in the child's life, not only in his or her medical condition. This is confirmed in earlier research (Ambresin, Bennett, Patton, Sanci, & Sawyer, 2013; Beresford & Sloper, 2003; Lugasi, Achille, & Stevenson, 2011; Pelander, Lehtonen, & Leino-Kilpi, 2007; Schmidt et al., 2007; Sällfors & Hallberg, 2009). If healthcare professionals have limited time, decision making must be as swift as possible, which inhibits a trustful relationship and the children's possibilities for participating in decision making (Virkki et al., 2014). Most healthcare situations generate stress, thus gentle movements, low voices, and calm surroundings are important for comforting children during medical procedures (Stephens, Barkey, & Hall, 1999). In accordance with our results, concerning participation, hospitalized children also express the need for comfort and stress-reducing places with color, windows, and playrooms (Norton-Westwood, 2012). The design of healthcare environments needs to be more adapted in order to help patients of all ages feel comfortable (Malenbaum, Keefe, Williams, Ulrich, & Somers, 2008; McCullough, 2010). The design of a hospital environment is important for reducing anxiety and fear in children (Norton-Westwood, 2012) as well as increasing their possibilities for participation (Stegenga & Ward-Smith, 2008). The importance of being surrounded by a sense of security and comfort, as expressed in the present study, is not explicitly described as important for children's participation in “the pathways to participation model” (Shier, 2001). One explanation could be that this model was developed for children in a community setting and not for children in healthcare environments. However, a trustful relationship with healthcare professionals is a prerequisite for increasing children's opportunities to be active in their own care. The process of transferring into the independence of adulthood has to start early for children with chronic diseases (Lugasi et al., 2011; Van Staa, Jedeloo, Van Meeteren, & Latour, 2011). The course of this process depends on the healthcare professional's ability to involve children in their own care. Children who have the possibility of being active and are allowed to plan their own care have been shown to attain greater self-determination and power (Aujoulat, Simonelli, & Deccache, 2006), as well as better health outcomes (Alderson, Sutcliffe, & Curtis, 2006). The multidirectional process described in the results section of this study can be used in clinical settings as a foundation for creating strategies to promote children's participation in healthcare. Future research is needed to understand how this result can be implemented into clinical practice across pediatric care settings and populations.

## Methodological considerations

By using an explorative qualitative design on the basis of the constructivist grounded theory (Charmaz, 2014), greater understanding has been gained concerning the experiences and preferences that promote participation in healthcare situations among children with JIA. In a constructivist grounded theory study, trustworthiness is established by credibility, resonance, usefulness, and originality. The credibility of the present study was strengthened by using a variety of methods to collect data, including individual interviews, focus groups, and different interview techniques, such as draw-and-tell and role play with dolls. The individual interviews provided rich data, while the focus groups inspired children to recall situations they might have forgotten. The sample size was not predetermined but depended on the ability of the participants to provide rich data. In order to strengthen the resonance, two authors began to analyze the data separately and then compared the concordance of the results. The credibility was further strengthened by systematic comparisons between collected data and the initial emerging categories. These comparisons are essential in the constructivist grounded theory method (Charmaz, 2014) in order to enhance and confirm the result of new data that emerges. The categories in our study portray the essential experiences and preferences described by the participants on how to promote participation in healthcare situations, strengthening the resonance of the result.

In a constructivist grounded theory study, the researchers interpret and construct the data. Moreover, since they are a part of the world they study, their experiences cannot be ignored but are used as a resource in the interpretation of the data (Charmaz, 2014). The thorough description of the children, the data collection, and the analysis enables the readers to determine the usefulness of the results for children with other diseases or in other contexts. The presentation of the results, together with appropriate quotations, enhances their usefulness. The originality was strengthened by the absence of any other study which had investigated children's perspectives on factors that promote participation of children with chronic diseases in their healthcare. The new knowledge gained from the present study may contribute to increase the ability of children with JIA or other chronic diseases to be more involved in healthcare situations.

## Conclusion

This study resulted in the core category, “releasing fear and uncertainty opens up for confidence and participation,” describing the processes that can influence the promotion of children's participation in healthcare situations from the children's perspective. The promotion of children's participation is a multidirectional process in which the categories “being surrounded by a sense of security and comfort” and “being strengthened and supported to become involved” are essential for healthcare professionals who wish to develop strategies for the promotion of children's participation in practice. Important aspects are the attitude and treatment by the healthcare professionals and child-adapted healthcare environments that make it possible for children to gain a sense of control over their situation and increase their self-confidence. The knowledge gained in this study provides new insights from the perspective of children themselves, and can constitute a valuable contribution to the understanding of the conditions necessary for the development of specific interventions that promote participation among children in healthcare situations.
